# ﻿Re-assessment of type material of *Plagiotheciumnovae-seelandiae* Broth. and descriptions of four new *Plagiothecium* taxa (Bryophyta, Plagiotheciaceae) from Australasia

**DOI:** 10.3897/phytokeys.238.114303

**Published:** 2024-02-09

**Authors:** Grzegorz J. Wolski, Mikołaj Latoszewski, D. Christine Cargill, William R. Buck

**Affiliations:** 1 University of Lodz, Faculty of Biology and Environmental Protection, Department of Geobotany and Plant Ecology, Banacha St. 12/16, 90-237 Lodz, Poland University of Lodz Lodz Poland; 2 Australian National Herbarium, Centre for Australian National Biodiversity Research (a joint venture between Parks Australia and CSIRO), GPO, Box 1700 Canberra, ACT 2601, Australia Australian National Herbarium, Centre for Australian National Biodiversity Research Canberra Australia; 3 Institute of Systematic Botany, The New York Botanical Garden, Bronx, New York 10458-5126, USA Institute of Systematic Botany, The New York Botanical Garden New York United States of America

**Keywords:** Australia, new taxa, New Zealand, *Plagiotheciumcordatum*, *P.novae-seelandiae* var. *brotheri*, *P.semimortuum*, *P.semimortuum* var. *macquariense*, taxonomic revision

## Abstract

A re-examination of the original collection of *Plagiotheciumnovae-seelandiae* described by Brotherus in 1916 indicated that this material is not homogeneous. Re-examination of the diagnosis of this species and morphological analysis supports that two separate taxa should be distinguished – Plagiotheciumnovae-seelandiaevar.novae-seelandiae and P.novae-seelandiaevar.brotheri**var. nov.** Also, comparisons with the original collection of *Hypnumlamprostachys* (= *P.lamprostachys*) showed differences, which supported their treatment as separate taxa. Revision of the genus *Plagiothecium* from Australasia (CANB, CHR, HO, MEL, WELT) and types of other species described from this part of the world (*P.funale* and *P.lucidum*) supported by the study of their diagnoses, qualitative and quantitative characteristics as well as mathematical analyses (PCA, HCA) allowed the division of the examined material into six separate groups – six separate taxa. Thereby, three distinct taxa are proposed – *P.cordatum***sp. nov.**, *P.semimortuum***sp. nov.**, and P.semimortuumvar.macquariense**var. nov.** All taxa mentioned above are described in detail, their current known distribution and ecological preferences are also included. In addition, images illustrating their most important taxonomic features, as well as an original key to distinguish individual taxa are presented.

## ﻿Introduction

In terms of species richness within the genus *Plagiothecium* Schimp., Australasia, comprised of Australia and New Zealand (Deverson 2005), is the most depauperate region in the world. Since the beginning of bryological research in this region of the world, only eleven names related to the described genus have appeared (Mitten 1856, 1882; Wilson 1859; Jaeger and Sauerbeck 1876–1877; Brotherus 1916; Ireland 1992; Ochyra 2002; Wynns 2015; Wynns et al. 2018). This low number of taxa compared to other regions of the world is possibly a result of relatively few revisions and the many morphological complexities associated with the genus (Ireland 1992; Ochyra 2002). Moreover, this fact may probably be influenced by geology, geography, biotic and abiotic factors, so it is difficult to assess this fact at this stage.

The earliest references to *Plagiothecium* in Australasia were from Tasmania and New Zealand concern *P.denticulatum* (Hedw.) Schimp., then known as a *Hypnumdenticulatum* Hedw. (Mitten 1856; Wilson 1859). Several years later, Hampe (1860) described a new species, *H.lamprostachys* Hampe, which is now known as *Plagiotheciumlamprostachys* (Hampe) A.Jaeger (Jaeger and Sauerbeck 1876–1877). Over the next few decades, this taxon was documented from other parts of Australia (Mitten 1882; Weymouth 1894; Rodway 1914, 1916). During this same period, Brotherus (1916) published the new species, *P.novae-seelandiae* Broth.

At the turn of the 20^th^ century there appeared a number of names that were incorrectly published or have been transferred to other genera: *P.amblystomum* Müll.Hal., *nom. nud*., *P.howei* Kindb. *nom. nud.*, and *P.novae-valesiae* Broth. are synonymous with *Ectropotheciumnovae-valesiae* (Broth.) Ireland (Ireland 1992); *P.howeanum* A.Jaeger, *nom. nud.* is synonymous with *Ectropotheciumleucochlorum* (Hampe) Broth.; and *P.helvolum* Müll.Hal., *in herb.* is synonymous with *Saulomatenella* (Hook.f. & Wilson) Mitt. (Fife 2019).

In the first decades of the 20^th^ century, the perception of the genus *Plagiothecium* in Australasia was greatly influenced by the publications of Dixon (1929) who recognized *P.denticulatum* and *P.novae-seelandiae* for New Zealand. At the same time, he treated *P.lamprostachys* as a synonym of *P.denticulatum* and in relation to the latter indicated that “the (...) status of *P.novae-seelandiae* therefore, is open to question.” Probably for this reason, in this part of the world *P.lamprostachys* was forgotten for many decades, and in later studies *P.novae-seelandiae* was reduced to a synonym of *P.denticulatum* (e.g., Sainsbury 1955). Thus, for the ensuing years, *P.denticulatum* was reported as the only representative of this genus (e.g., Sainsbury 1955; Scott and Stone 1976; Ramsay 1984; Streimann and Curnow 1989; Beever et al. 1992) for Australasia. However, Sainsbury (1955) did indicate the remarkable variability of this taxon. By the end of the 20^th^ century *Plagiotheciumlaetum* Schimp. was also recognized for the area (Vitt 1974), but later the presence of this typical Northern Hemisphere taxon was questioned by Fife (2019), and subsequently excluded from the flora of Australasia.

The end of the 20^th^ century sees the revision by Ireland (1992), which shed new light on the perception of the genus *Plagiothecium* in Australasia. This researcher stated that there is a significant difference between *P.denticulatum* and *P.novae-seelandiae*, proposing the resurrection of the latter, as a separate species and deletion of *P.denticulatum* from the moss flora of Australasia. Additionally, Ireland (1992) published the first occurrence of *Plagiotheciumlucidum* (Hook.f. & Wilson) Paris from Australia and New Zealand. Ten years later, the presence of *P.lucidum* in Australasia was confirmed by Ochyra et al. (2000).

The beginning of the 21^st^ century brings Ochyra’s (2002) publication in which he indicated that plants in the original collections of *P.lamprostachys*, in terms of leaf shape and general habit, match perfectly the type collections of *P.novae-seelandiae*. Thus, he proposed to synonymize the latter with *P.lamprostachys* (Ochyra 2002). This point of view was almost immediately adopted by all Australasian bryologists (e.g., Streimann and Klazenga 2002; Klazenga 2012; Seppelt et al. 2013; Fife 2019). Streimann and Klazenga (2002), like Ireland (1992) and Ochyra et al. (2000), also additionally reported *P.lucidum*. A few years later appears a review by Wynns (2015) and Wynns et al. (2018). Wynns (2015) not only understood *P.lamprostachys* and *P.novae-seelandiae* as separate taxa, but also described two new species from this area in his Ph.D. thesis – *P.funale* J.T.Wynns and *P.humile* J.T.Wynns. However, in a later study (Wynns et al. 2018), *P.humile* was no longer distinguished and was omitted. We have been unable to find material which formed the basis of *P.humile*, *nom. inval*.

The complicated taxonomic history and relatively small number of species was the impetus to provide a revision of the genus for Australasia with the aim of testing the assumptions and taxonomic concepts presented by previous researchers.

## ﻿Materials and methods

All collections of the genus *Plagiothecium* deposited in CANB, CHR, HO, MEL, and WELT– almost 400 specimens – were examined. After the revision, only those specimens with symmetrical leaves were selected for further analysis.

Thus, 27 specimens were selected, including four specimens (types) of *Hypnumlamprostachys* (=*Plagiotheciumlamprostachys*) (BM000677526!, BM000677527!, BM000677528!, NY322494!); two specimens (types) of *P.funale* (CHR267040!, MO2408073!); five specimens (types) of *P.novae-seelandiaesensu lato* (CHR534780!, CHR534781!, PC0132644!, PC0132645!, PC0132646!); three specimens of material later named *Plagiotheciumcordatum*, as well as 11 specimens of *Plagiotheciumsemimortuumsensu lato.* The two specimen types *P.lucidum* (PC0132689!, PC0132690!) were also analyzed. Thanks to this, all taxa described so far from Australasia were examined.

**Figure 1.**
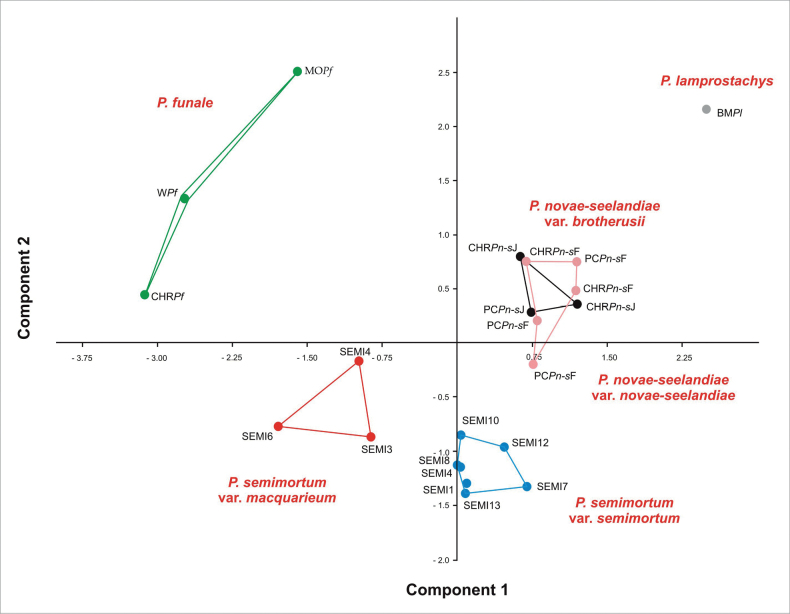
PCA analysis of the tested specimens. Explanation: CHR – Christchurch herbarium, PC – Paris herbarium, BM – Natural History Museum Herbarium, MO – Missouri herbarium, *Pn-s* – *Plagiotheciumnovae-seelandiae*, *Pl* – *Plagiotheciumlamprostachys*, *Pf* – *Plagiotheciumfunale*, F – complanate leaves, J – julaceaous leaves, W – data on *P.funale* based on literature analysis (Wynns 2015; Wynns et al. 2018), SEMI1, 10, 12, 13, 14, 17, 18 – P.semimortuumvar.semimortuum, SEMI3, 4, 6 – P.semimortuumvar.macquariense.

**Figure 2.**
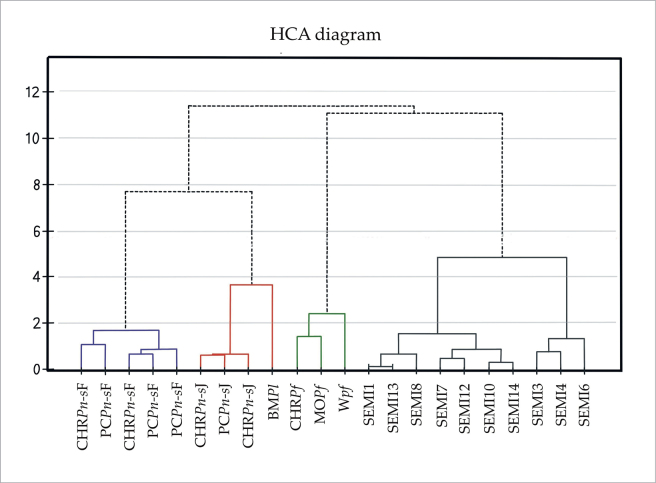
Dendrogram (HCA) of the examined specimens. Explanation: see Fig. 1.

Selected specimens were used not only for mathematical analyses, but also for the description of new taxa. The mathematical analyses were performed mainly on nomenclatural types of taxa previously known from Australasia and the similar but later-named *P.semimortuum* (Figs 1, 2).

The selection of features for the following study was made on the basis of methodology adopted by Wolski (2017, 2019) and Wolski and Nowicka-Krawczyk (2020). Thus, the features include not only the most taxonomically important ones, but also other characteristics basic to the description of individual taxa: qualitative and quantitative features of gametophytes and sporophytes of the examined plants. Therefore, color, luster, and habit were tested first. From a uniform turf, one stem was chosen; the length of the whole stem was measured, and the arrangement of the leaves on the stem was evaluated.

Then, all the leaves were torn off from the central part of the stem, and six leaves were randomly selected for further measurements. For each of the examined leaves, the shape, symmetry, folding, and concavity were evaluated. They were also measured in terms of the length and the width at their widest points and the length of both costae. Additionally, the shape, curvature, and serration of the leaf apex were observed.

For each of the selected leaves, five groups of cells were measured: from the upper, the middle and the lower part of the leaf. Laminal cell shape was assessed, additionally, alar cells were measured, and their shape was assessed. Decurrent leaf base cells were measured, and the number of rows of cells was counted. The cross-section was taken from the central part of the stem and six cross-sections of the stems were randomly chosen. First, the diameter of the obtained stem cross-section was measured, then five epidermal cells and five parenchymal cells were randomly selected.

In addition, the length of the sporophyte was assessed, color of the seta, length and width of the capsules, its arrangement on the seta, shape and length of operculum – of course only if these elements were present in the material. Similarly, in the case of other features – they were omitted from the description when a given element was not present or the feature was impossible to determine. Due to the poor condition of specimens, this situation occurred in the case of some gametophytic features of *P.lamprostachys*. Moreover, sporophytes were missing for P.novae-seelandiaevar.brotheri (PC0132644, CHR534780), *P.cordatum* (CHR538916) and P.semimortuumvar.macquariense (HO610220).

All research in the presented manuscript was based on our own macroscopic and microscopic analysis of herbarium collections. Only in one case, and only for the purposes of the cluster analyses (Figs 1, 2), was data used based on *P.funale* literature (Wynns 2015; Wynns et al. 2018). However, when describing this species, only data collected from the analysis of herbarium specimens were taken into account (MO2408073, CHR267040).

On the basis of features recognized in the literature as the most taxonomically important — length and width of leaf, length and width of cells from midleaf (e.g., Wolski and Nowicka-Krawczyk 2020; Wolski et al. 2021) — grouping analyses of the studied taxa were carried out. Due to the incommensurability of the data (length and width of leaf to length and width leaf cell), Principal Component Analysis (PCA) and Hierarchical Cluster Analysis (HCA) were used to arrange the points in the ordering space.

These analyses are a basic tool that allows for grouping the examined specimens and thus showing the similarity between them. All mathematical analyses were performed in the PQSTAT v. 1.8.6 program. All other above-mentioned features considered representative of this genus were used to describe individual taxa (e.g., Wolski 2017, 2019; Wolski, Nowicka-Krawczyk 2020; Wolski et al. 2022a, b).

## ﻿Results

**Figure 3.**
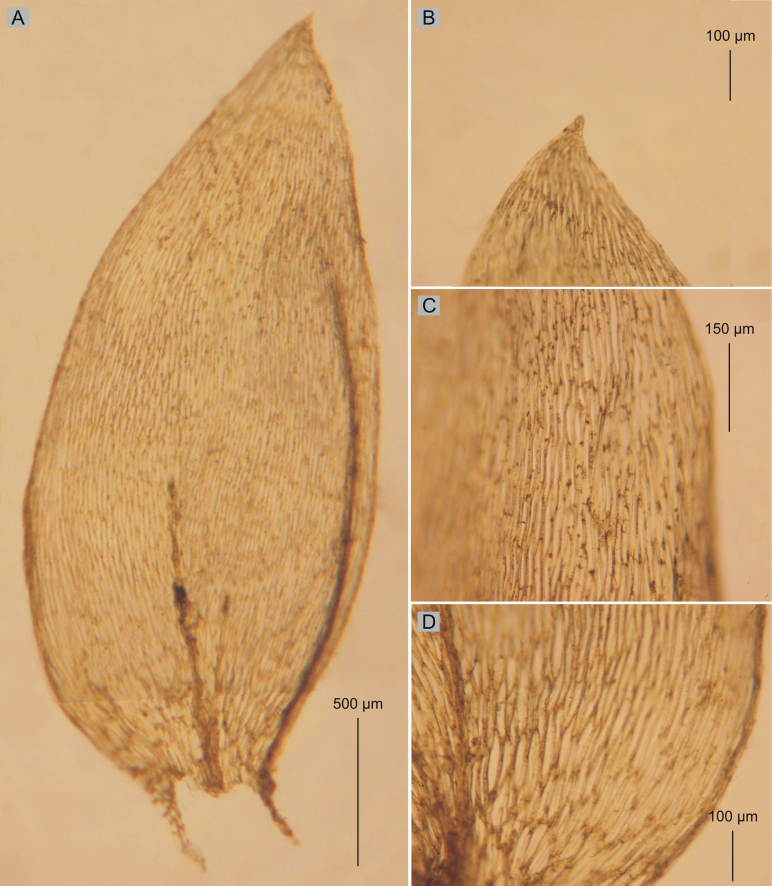
Selected taxonomic features of *P.lamprostachys***A** shape and dimensions of the leaf **B** leaf apex **C** cells from the middle part of the leaf **D** leaf basal cells (from the type of material of *H.lamprostachys* BM000677528!, photo. G. J. Wolski, 01 August 2023).

**Figure 4.**
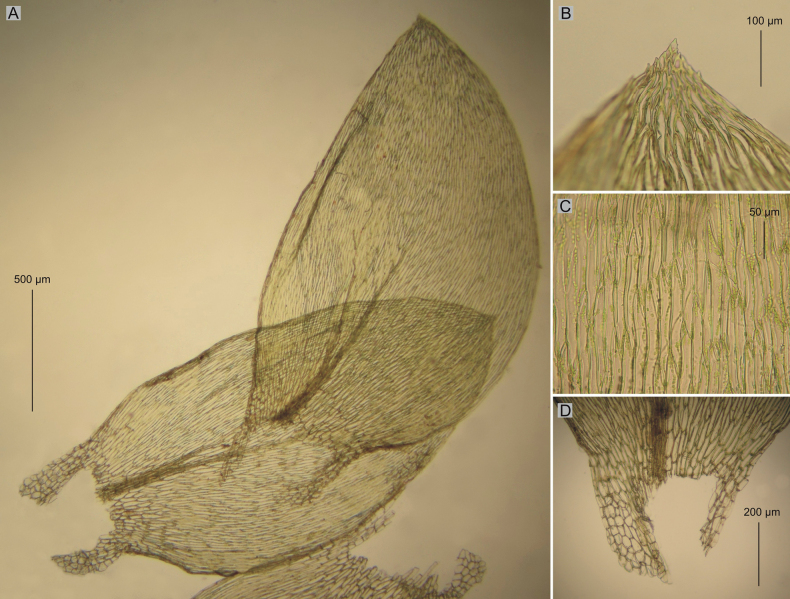
Selected taxonomic features of Plagiotheciumnovae-seelandiaevar.novae-seelandiae**A** shape and dimensions of leaves **B** serrate leaf apex **C** dimensions and shape of cells from middle part of the leaf **D** decurrency (from the type material of *P.novae-seelandiae* PC0132644p.p.!, photo. G. J. Wolski, November 21, 2021).

**Figure 5.**
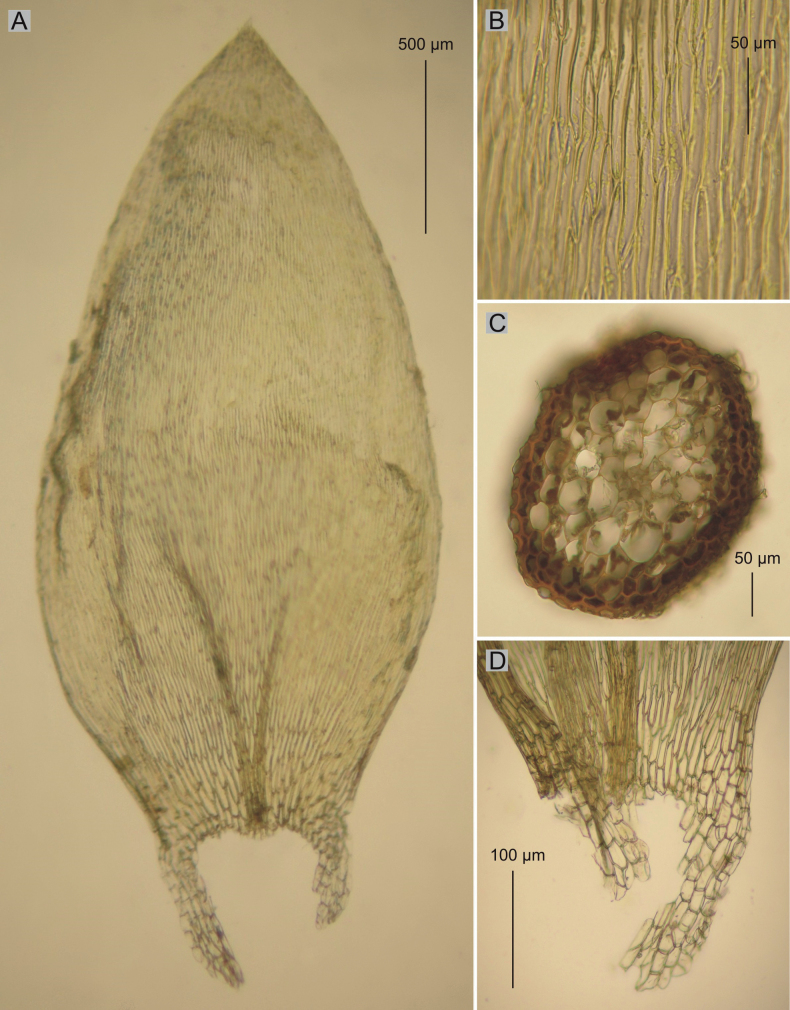
Selected taxonomic features of Plagiotheciumnovae-seelandiaevar.brotheri**A** shape and dimensions of leaf **B** dimensions and shape of cells from middle part of the leaf **C** stem cross-section **D** decurrency (from the type material of *P.novae-seelandiae* PC0132644p.p.!, photo. G. J. Wolski, November 22, 2021).

**Figure 6.**
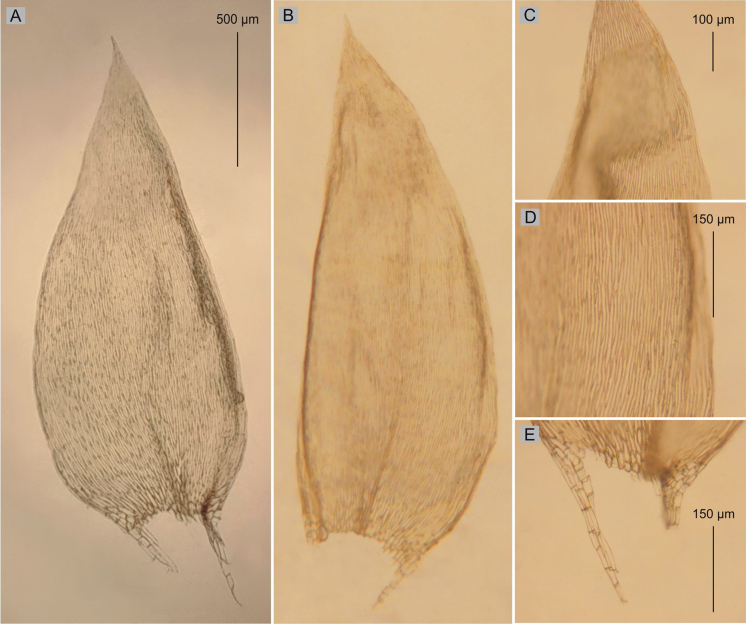
Selected taxonomic features of *Plagiotheciumfunale***A, B** shape and dimensions of leaves **C** folding of the apex of the leaf **D** dimensions and shape of cells from middle part of the leaf **E** narrow decurrency composed of rectangular cells (from the type of material of *P.funale* CHR267040!, MO2408073!, photo. G. J. Wolski, November 2022 and July 2023).

**Figure 7.**
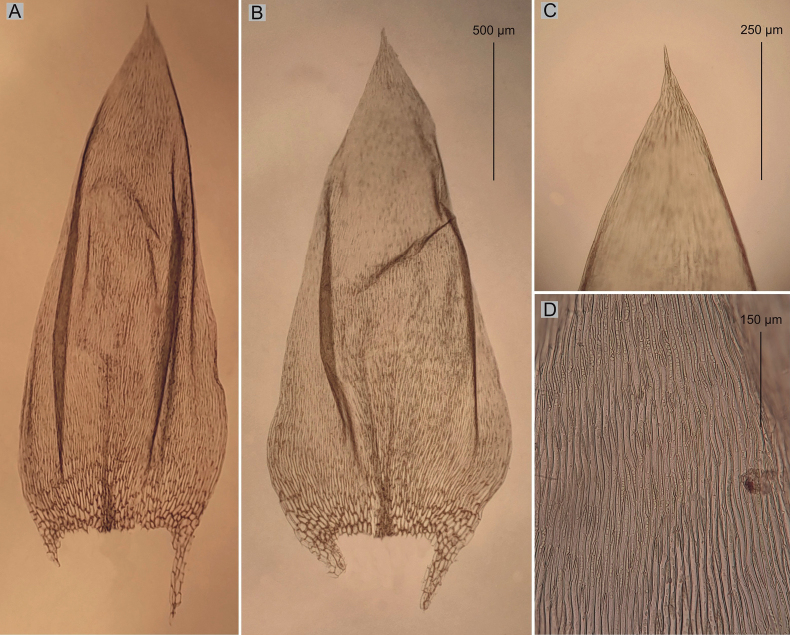
Selected taxonomic features of *Plagiotheciumcordatum***A, B** shape and dimensions of leaves **C** leaf apex **D** dimensions and shape of cells from middle part of the leaf (from the type of material of *P.cordatum* CHR538916!, photo. G. J. Wolski, November 12, 2022).

**Figure 8.**
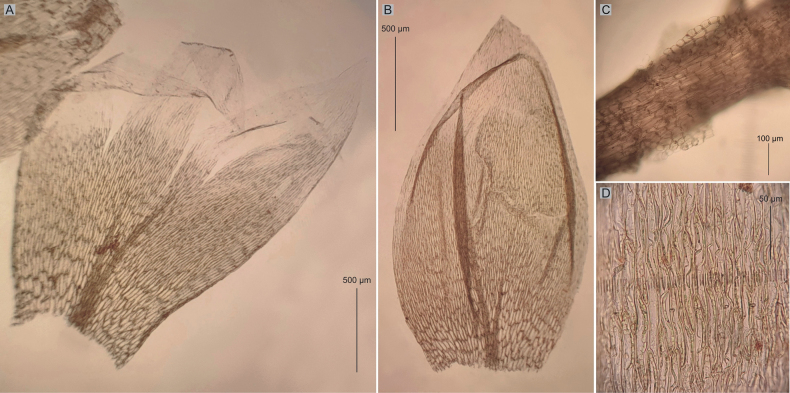
Selected taxonomic features of Plagiotheciumsemimortuumvar.semimortuum**A, B** shape and dimensions of leaves **C** decurrency on the stem **D** dimensions and shape of cells from middle part of the leaf (from the type of material of Plagiotheciumsemimortuumvar.semimortuum MEL1016042 and WELT-M28128, photo. G. J. Wolski, November 13, 2022).

**Figure 9.**
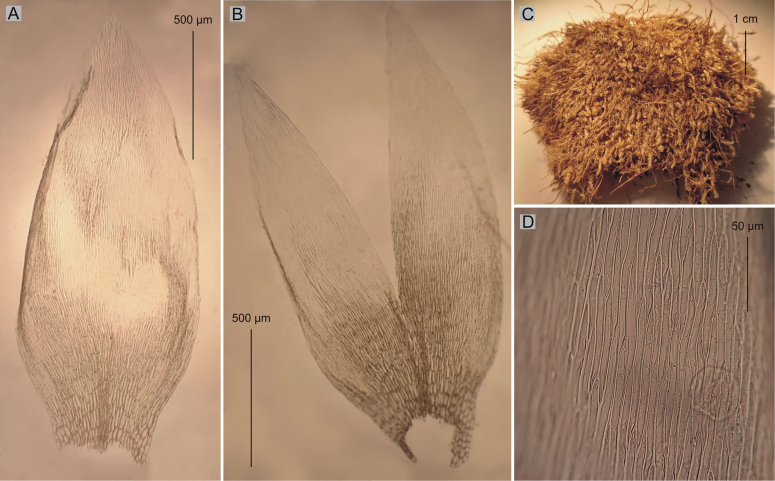
Selected taxonomic features of Plagiotheciumsemimortuumvar.macquariense**A, B** shape and dimensions of leaf **C** julaceous turf **D** dimensions and shape of cells from middle part of the leaf (from the type of P.semimortuumvar.macquariense HO610220, photo. G. J. Wolski, November 13, 2022).

**Figure 10.**
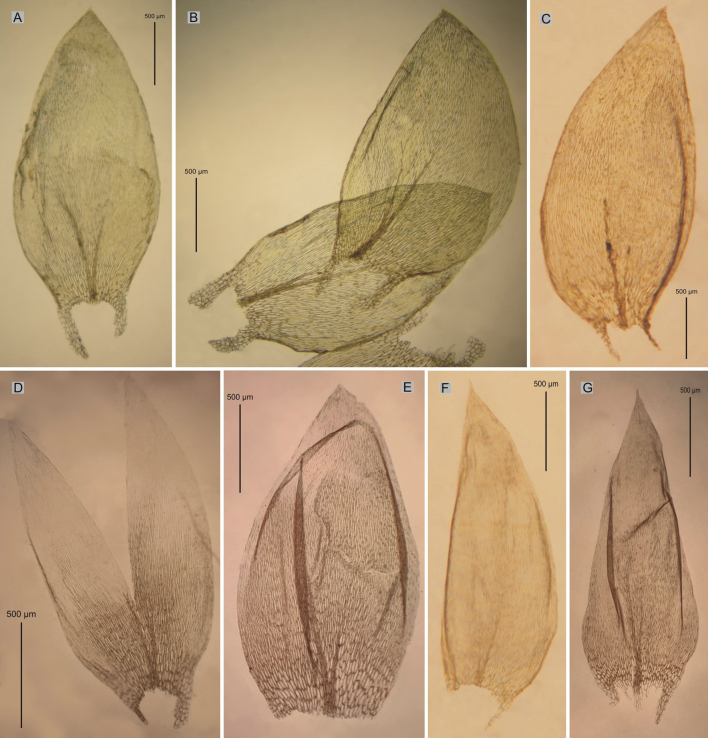
Comparison of leaf shapes and dimensions of all described taxa **A**P.novae-seelandiaevar.brotheri**B**P.novae-seelandiaevar.novae-seelandiae**C***P.lamprostachys***D**P.semimortuumvar.macquariense**E**P.semimortuumvar.semimortuum**F***P.funale***G***P.cordatum* (based on the types of the above-mentioned taxa, see Figs 3–9).

**Figure 11.**
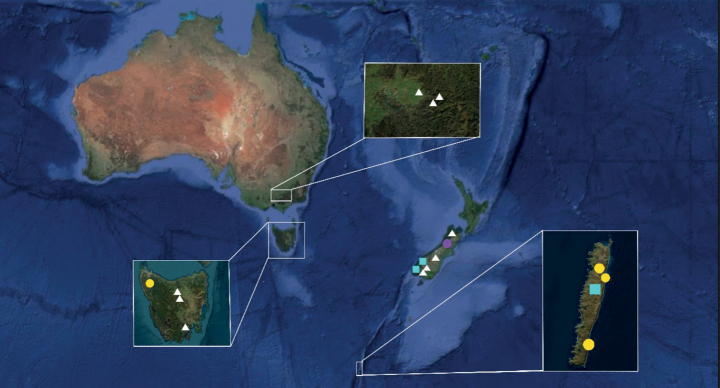
Distribution of the newly described taxa. Explanation: white triangles – P.semimortuumvar.semimortuum; yellow circles – P.semimortuumvar.macquariense; purple circles – P.novae-seelandiaevar.brother; aquamarine squares – *P.cordatum* (Google Maps, accessed September 15, 2023).

The analyzed types as well as other material of *Hypnumlamprostachys* (=*Plagiotheciumlamprostachys*) (BM000677526!, BM000677527!, BM000677528!, NY322494!), *P.novae-seelandiae* (CHR534780!, CHR534781!, PC0132644!, PC0132645!, PC0132646!), and *P.funale* (CHR267040!, MO2408073!) showed remarkable heterogeneity, wherein two separate morphotypes have been distinguished within *P.novae-seelandiae*. They differ both in several qualitative and quantitative features (Figs 1–11). Thus, the PCA and HCA analysis shows the division of the examined specimens and their grouping into five separate groups, with one of them showing internal differentiation (Figs 1, 2). In PCA, individual axes explain in total 76.9% of the variability (the first axis 45.7%, the second axis 31.2%) (Fig. 1).

Among the studied materials, the first group consists of the types of *Hypnumlamprostachys* (= *Plagiotheciumlamprostachys*) and *P.novae-seelandiae*, wherein the *P.lamprostachys* specimens (BM000677526!, BM000677527!, BM000677528!, and NY322494!) stand out, clearly different from the other specimens. *Plagiotheciumlamprostachys* material is characterized by asymmetric or slightly asymmetrical, long, broad (2.5–2.6 × 1.0–1.2 mm), ovate, concave leaves, apex entire, and long, broad laminal cells (140–150 × 12–13 µm) (Fig. 3). The other specimens (*P.novae-seelandiae*) form a non-heterogeneous group (Figs 1, 2).

*Plagiotheciumnovae-seelandiae* was described by Brotherus in 1916. In the diagnosis, the author indicated that the specimen is densely foliate, more or less complanate-foliate, the leaves are concave, long-decurrent, broadly ovate, asymmetrical, with elongate, loosely rhomboidal cells (Brotherus 1916). Examination of isolectotypes of *P.novae-seelandiae* (CHR534780!, CHR534781!, PC0132644!, PC0132645!, PC0132646!) showed that this material is not homogeneous, but is a mixture of two different morphotypes (Figs 1, 2, 4, 5). The existence of two groups of morphotypes within *P.novae-seelandiae* is confirmed by the mathematical analyses performed. However, the overlap of these groups in the PCA analysis is only related to the two-dimensional possibility of showing the results, and the distinctiveness of the above-mentioned groupings is confirmed by the HCA analysis (Figs 1, 2).

One of the morphotypes (CHR534781!, CHR534780p.p.!, PC0132644p.p.!, PC0132645!, PC0132646!, H3301105, available online!) with complanate stems, is characterized by a dominance of asymmetric leaves, serrate leaf apices, wide cells, making the cell areolation very loose (100–130 × 12–17 µm). This description fits very well with the diagnosis of *P.novae-seelandiae* given by Brotherus (1916). The second morphotype (CHR534780p.p.!, PC0132644p.p.!) is characterized by julaceous stems, a dominance of symmetrical leaves, entire, non-serrate leaf apices, narrow cells, making the cell areolation tight (100–140 × 7.5–10 µm) (Figs 4, 5). Specimens of *P.novae-seelandiae* with julaceous stems (CHR534780p.p.!, PC0132644p.p.!) differ from *P.lamprostachys* in habit, shape, symmetry and size of the leaves (Figs 1–3) as described above.

Taking into account the above facts, it can be indicated that plants with complanate-foliate, asymmetric leaves, serrate apices, wide cells, making the cell areolation loose refer to *P.novae-seelandiae* which was described by Brotherus (1916) (Fig. 4). The second morphotype, refers to the new variety proposed here – P.novae-seelandiaevar.brotheri (Fig. 5).

Another group of specimens are material representing *P.funale* (Figs 1, 2). They differ from other examined specimens by leaves loosely arranged on the stem, lanceolate, clearly asymmetric, short and narrow (1.6–2.2 × 0.6–0.8 mm), concave, folded, short leaves, elongate and entire, non-serrate apices, long and narrow laminal cells (120–150 × 6–7 µm) and wedge-shaped, narrow decurrencies composed of rectangular cells (Fig. 6).

Narrow decurrencies are a feature that distinguishes *Plagiotheciumfunale* from other taxa of this genus with wide decurrencies, encompassing all those taxa currently known from Australasia. However, the analysis also indicated the presence of another taxon with narrow decurrencies, distinguished by julaceous stems, short and narrow (1.7–2.0 × 0.7–0.9 mm), longitudinally folded, concave, lanceolate, symmetric leaves with heart-shaped leaf bases, entire, non-serrate leaf apices, and long and narrow cells (140–165 × 5–7 µm), making the cell areolation tight. Specimens with such features (Fig. 7) we propose to call *Plagiotheciumcordatum* sp. nov.

The last two taxa are plants with a unique set of gametophytic qualitative and quantitative features (Figs 8, 9). The unique feature, otherwise not found among taxa of this genus, is the absence of protoplast in the upper part of the leaf at maturity.

The first group (SEMI1, SEMI13, SEMI8, SEMI7, SEMI12, SEMI10 and SEMI14) (Figs 1, 2, 8) is material with erect, julaceous stems, symmetrical, concave, transversely undulate leaves, with a leaf from 1/3 to 2/3 devoid of protoplasts, short and wide laminal cells (60–90 × 10–12 µm), which makes the cell areolation very loose, and a decurrency constructed of spherical and inflated cells (Fig. 8). Specimens with such characteristics we propose to name here *Plagiotheciumsemimortuum* sp. nov. The second group of specimens (SEMI3, SEMI4, SEMI6) (Figs 1, 2, 9) differs from the previous one in narrower leaves, longer and narrower laminal cells (112.5–125 × 7.5–10 µm) and a different habitat – lowland areas. Specimens with such features (Fig. 9) we propose here to call Plagiotheciumsemimortuumvar.macquariense var. nov.

## ﻿Discussion

The genus *Plagiothecium* in Australasia has been misunderstood, and perceptions have changed considerably. First, practically all specimens from this part of the world were identified as *P.denticulatum*, then *P.novae-seelandiae*, and later *P.lamprostachys*. Thus, a single taxon name was replaced by successive names without a careful and detailed revision of the group (e.g., Mitten 1856, 1882; Wilson 1859; Brotherus 1916; Ireland 1992; Ochyra 2002). This is one of the reasons for the low number of taxa reported so far from Australasia. The second important contributing factor to the low number of taxa was the relatively small number of studies on this genus (Ireland 1992; Ochyra 2002; Wynns 2015; Wynns et al. 2018). Thus, for such a huge and diverse continent, the number of taxa recorded until the beginning of the 21^st^ century is extremely low, especially in comparison with other parts of the world, not just the relatively well-studied Northern Hemisphere (e.g., Nyholm 1965; Buck and Ireland 1989; Smith 2001; Wolski et al. 2021).

Dixon (1929) was a big influence on the perception of this genus, for example, his synonymization of *P.lamprostachys* with *P.denticulatum* led to the loss of the concept of that species for decades. A similar influence was Sainsbury (1955), who synonymized *P.novae-seelandiae* with the aforementioned *P.denticulatum*. On the other hand, the confusion of Australian and New Zealand specimens with *P.denticulatum* is not so surprising. Because both the habit and the most important microscopic features (e.g., serrate leaf apex, loose areolation of cells, distinct decurrency composed of inflated cells) resemble this most common Northern Hemisphere taxon (Wolski et al. 2021). However, as in many other cases (e.g., *Plagiotheciumschofieldii* G.J.Wolski & W.R.Buck and *P.lamprostachys*), despite the morphological similarity, geographical and molecular differences between them are indisputable (Wynns et al. 2018; Wolski et al. 2021), as are subtle morphological differences.

Although *P.denticulatum* has been reported from Australasia for decades, Ireland (1992) rightly excluded it from the local moss flora. As in the case of *P.denticulatum*, the same was with *P.laetum* (Vitt 1974). This species was most likely confused by Vitt with the similar *P.lucidum*, in terms of leaf symmetry and cell dimensions. However, as in the case of *P.denticulatum*, we now know that *P.laetum* is a taxon occurring only in the Northern Hemisphere, and thus was excluded from Australasia by Fife (2019).

Ochyra (2002) indicated that the types of *P.lamprostachys* match perfectly to the type collections of *P.novae-seelandiae*. This synonymization further influenced the understanding and perception of this genus in Australasia. Current research indicates that indeed one of the morphotypes of *P.novae-seelandiae* is similar to *P.lamprostachys*, but they are not identical, which is indicated by the statistical analyses. Treating these species separately was already proposed by Wynns (2015), and it is supported here.

Interestingly, none of the earlier researchers (e.g., Ireland 1992; Ochyra 2002; Fife 2019) indicated that the original collection of *P.novae-seelandiae* consisted of two separate morphotypes. Only Wynns (2015) mentioned it, but generally ignored the issue. However, even a cursory analysis signifies that the two previously mentioned morphotypes of *P.novae-seelandiae* differ in many important taxonomic features – e.g., habit, foliage, symmetry, concavity of leaf, serration of apex, cell dimensions. These differences are supported by the statistical analyses presented above. Morphological studies combined with the analysis of the diagnosis indicate that the taxon mentioned by Brotherus (1916) is material characterized by, for example, complanate-foliate habit, domination of asymmetrical leaves, serrate leaf apices, wide cells, which makes the cell areolation loose (100–130 × 12–17 µm). Specimens with such a set of features were named *Plagiotheciumnovae-seelandiae* [var.novae-seelandiae] (CHR534781!, CHR534780p.p.!, PC0132644p.p.! PC0132645!, PC0132646!). But, the morphotype with julaceous stems, dominance of symmetrical leaves, entire, non-serrate apices, narrow cells, which makes the cell areolation tight (100–140 × 7.5–10 µm) is here named Plagiotheciumnovae-seelandiaevar.brotheri.

Wynns (2015), in his doctoral thesis, described two new species from Australasia – *Plagiotheciumfunale* and *P.humile*. However, in his publication based on his thesis (Wynns et al. 2018), he does not mention the latter. On the other hand, the former – *P.funale* can be distinguished easily from the other taxa recorded currently from Australasia by asymmetrical, concave, undulate leaves, short and smooth apex, long and narrow cells and wedge-shaped, and narrow decurrencies composed of rectangular cells. The last feature – wedge-shaped decurrencies, composed of rectangular, non-inflated cells – is a very important and the most unique feature compared to other species. Decurrent angular rounded cells forming distinct auricles are characteristic of all previous species (e.g., Sainsbury 1955; Scott and Stone 1976; Ireland 1992; Fife 2019; Ochyra 2002).

In the genus *Plagiothecium*, the decurrency is one of the most important taxonomic features (Wolski et al. 2021). Although this feature plays a fundamental role in the division of individual taxa into sections of this genus, unfortunately it is often overlooked when analyzing material – therefore, it is always necessary to analyze these structures, which in the case of this genus often remain on the stem after dissection. Without checking the decurrency, it is very easy to confuse some even distantly related species that are similar in some respects, e.g., *P.denticulatum* and *P.nemorale* (Mitt.) A.Jaeger (e.g., Wolski and Nowicka-Krawczyk 2020; Wolski et al. 2021).

*Plagiotheciumcordatum*, like *P.funale*, is characterized by a unique set of gametophyte features, including, and most importantly, a wedge-shaped decurrency composed of uninflated cells (Wynns 2015; Wynns et al. 2018). This feature distinguishes these taxa from other taxa in this part of the world. The other characteristics, e.g., julaceous stem, short and narrow (1.7–2.0 × 0.7–0.9 mm), concave, clearly and strongly folded leaves with a heart-shaped base, entire, non-serrate leaf apices, and long and narrow cells (140–165 × 5–7 µm) make it quite easy to distinguish *P.cordatum* from *P.funale*.

This research has also allowed the description of Plagiotheciumsemimortuumvar.semimortuum and P.semimortuumvar.macquariense. Both have a unique feature not found in any other species of the genus. The leaf cells are devoid of protoplasts occupying as much as 2/3 of the leaf length. The absence of the protoplasts in part of the leaf is unusual for the genus *Plagiothecium* (Smith 2001; Wolski et al. 2021), but not for some types of mosses. Many taxa, especially those growing in open, sunny habitats, are characterized by a lack of protoplasts in the leaf or part of the leaf, e.g., *Bryumargenteum* Hedw., *Gigaspermummouretii* Corb., *Orthotrichumdiaphanum* Brid., *Polytrichumpiliferum* Hedw., *Tortulamuralis* Hedw. and others (e.g., Noguchi 1995; Smith 2001).

These two taxa, Plagiotheciumsemimortuumvar.semimortuum and P.semimortuumvar.macquariense, due to the decurrent angular rounded cells, which form distinct auricles clearly have been referred to *P.lamprostachys* and *P.novae-seelandiaesensu lato* (Sainsbury 1955; Scott and Stone 1976; Ramsay 1984; Ireland 1992; Fife 2019; Ochyra 2002), and because of this morphology, they should be included in Plagiotheciumsect.Plagiothecium.

Despite some similarities, P.semimortuumvar.semimortuum and P.semimortuumvar.macquariense differ in a number of qualitative and quantitative gametophytic features: the size and folding of the leaf, the serration of the leaf apex, the dimensions of the cells, but also the habitat – mountains versus lowlands. All these features confirm the validity of distinguishing the above-mentioned taxa.

### ﻿Taxonomic treatments

#### ﻿ Plagiothecium lamprostachys

Taxon classificationPlantaeHypnalesPlagiotheciaceae

(Hampe) A.Jaeger, Bericht über die Thätigkeit der St. Gallischen Naturwissenschaftlichen Gesellschaft 1876–1877: 449 (1878)

8B145EF4-3193-54E9-95B5-A8E712225A98

Hypnum lamprostachys Hampe, Linnaea 30: 639 (1860).

##### Type.

Australia, Hab. ad fl. Tarwin. ***Lectotype*** (selected by Ochyra 2002): Austral felix Tarwin, Herb. Hamp. – 1881. *Hypnumlamprostachys* Hpe. leg. *F. Mueller N°59*, 1855 (BM-Hampe!). ***Isolectotypes***: (BM000677526!, BM000677527!, BM000677528!, NY322494!).

##### Description.

Plants medium size, yellowish to yellow-green, with metallic luster, forming dense mats; stems 1.5–2.5 cm long, in cross-section rounded, the central strand well-developed; leaves asymmetrical to almost asymmetrical, ovate, concave, rather imbricate and closely arranged on the stem, those leaves from the middle of stem 2.5–2.6 mm long and the width measured at the widest point 1.1–1.2 mm (Fig. 3); the apex acute, entire, not denticulate; costae two, extending usually to 1/2 of the leaf length; laminal cells more or less symmetrical, arranged in fairly even rows, 140–150 × 12–13 μm in the middle of leaves; due to the wide cells, cell areolation loose; decurrency of 4 rows of rounded and inflated cells, forming distinct auricles, 200–250 μm long; sporophytes so far unknown; sexual condition unknown.

*Plagiotheciumlamprostachys* type material was recorded near the Tarwin River in Australia (Hampe 1860).

#### ﻿ Plagiothecium novae-seelandiae var. novae-seelandiae

Taxon classificationPlantaeHypnalesPlagiotheciaceae

Broth., Proceedings of the Linnean Society of New South Wales 41: 594 (1916)

419EBCAA-0E88-5364-8067-640B35A4F1F8

##### Type.

New Zealand, Kelly’s Range, Kelly’s Creek, on dripping rocks, and at top of Otira Gorge, 2830 ft., damp rocks in scrub, leg. *T. W. Naylor Beckett.****Lectotype*** (selected by Ireland 1992): New Zealand, Mosses of Westland, Damp rocks in scrub at top of Otira Gorge, 2830 ft, *Plagiothecium Novae Seelandiae* Broth., leg. *T. W. Naylor Beckett 918*, 11 Feb. 1903 (H3301105, available online!). ***Isolectotypes***: (CHR534780p.p.!, PC0132645!, NY322492!, NY322493!). ***Syntypes***: New Zealand, Mosses of Westland, Kelly’s Creek, Kelly’s Range, on dripping rocks, *Plagiothecium Novae Seelandiae* Broth., leg. *T. W. Naylor Beckett 996*, 3 Feb. 1903 (CHR534781!, PC0132644p.p.!, PC0132646!, DUKE156811, S-B160226, UC1911437).

##### Description.

Plants medium size, green, with metallic luster, forming rather dense mats, complanate-foliate; stems 4–6 cm long, in cross-section rounded, 300–350 μm in diameter, the central strand well-developed; leaves asymmetrical, not overlapping on the stem to slightly imbricate, rather flat to undulate, sometimes one side of the leaf flat or folded over the rest of the leaf, leaves from the middle of stem 1.7–2.2 μm long and the width measured at the widest point 1.0–1.5 mm; the apex acute and denticulate; costae two, rather thick and strong, extending usually to ½ of the leaf length; laminal cells more or less symmetrical, the length and width variable but dependent on location: 110–140 × 10 μm at apex, 100–130 × 12–17 μm at midleaf, and 75–150 × 17.5–20 μm toward insertion; due to the wide cells, cell areolation loose; decurrency of 3–5 rows of rounded and inflated cells, forming distinct auricles, 200 μm long. Sporophytes 2.5–4.0 cm long, setae reddish-orange; capsules horizontal, 1.7–2.8 × 0.7–1.0 mm (Fig. 4); sexual condition unknown.

Plagiotheciumnovae-seelandiaevar.novae-seelandiae types were recorded from New Zealand, Kelly’s Range, Kelly’s Creek (CHR534781!, PC0132644p.p.!, PC0132646!, DUKE156811, S-B160226, UC1911437) and at top of Otira Gorge (H3301105, available online!, CHR534780p.p.!, PC0132645!, NY322492!, NY322493!), on dripping rocks (H3301105, available online!, CHR534780p.p.!, PC0132645!, NY322492!, NY322493!, CHR534781!, PC0132644p.p.!, PC0132646!, DUKE156811, S-B160226, UC1911437), damp rocks in scrub (H3301105, available online!, CHR534780p.p.!, PC0132645!, NY322492!, NY322493!).

#### ﻿ Plagiothecium novae-seelandiae var. brotheri

Taxon classificationPlantaeHypnalesPlagiotheciaceae

G.J.Wolski var. nov.

6A6044EE-BE1F-594D-8820-F5AEB022B6AE

##### Type.

***Holotype***: Mosses of Westland, New Zealand, on dripping rocks, Kelly’s Creek, Kelly’s Range, *Plagiothecium Novae Seelandiae* Broth., leg. *T. W. Naylor Beckett 996*, 3 Feb. 1903 (PC0132644p.p.!). ***Paratype***: Mosses of Westland, New Zealand, damp rocks in scrub at top of Otira Gorge, 2830 ft, *Plagiothecium Novae Seelandiae* Broth., leg. *T. W. Naylor Beckett 918*, 11 Feb. 1903 (CHR534780p.p.!).

##### Description.

Plants medium size, green, julaceus, with metallic luster; stems 3–4 cm, in cross-section rounded, 250–300 μm in diameter, the central strand well-developed; leaves symmetrical to almost symmetrical, imbricate, concave, ovate, slightly folded, leaves from middle of stem 1.7–2.4 mm long and width measured at widest point 0.9–1.0 mm; leaf margins recurved; the apex acuminate, not denticulate; costae two, rather thick and strong, extending usually to 1/3 of the leaf length; laminal cells more or less symmetrical, the length and width variable but dependent on location: 90–120 × 7.5–10 μm at apex, 100–140 × 7.5–10 μm at midleaf, and 100–125 × 10–12.5 μm toward insertion; due to relatively narrow, cell areolation quite tight; decurrency of 5–6 rows of rounded and inflated cells, forming distinct auricles, 200–250 μm long (Fig. 5); sporophytes so far unknown; sexual condition unknown.

Plagiotheciumnovae-seelandiaevar.brotheri type material was recorded from New Zealand, Kelly’s Creek, Kelly’s Range (PC0132644p.p.!) and at top of Otira Gorge (CHR534780!), on dripping rocks (PC0132644p.p.!) and damp rocks in scrub (CHR534780!).

##### Etymology.

The present taxon is part of the *P.novae-seelandiae* collection from which Brotherus (1916) described a new species, therefore this taxon — P.novae-seelandiaevar.brotheri — is named in honor of Brotherus.

#### ﻿ Plagiothecium funale

Taxon classificationPlantaeHypnalesPlagiotheciaceae

J.T.Wynns, Cladistics 34(5): 483. 2018 [11 October 2017]

4DD01951-7C21-5C24-AAA9-BBA11B05B06C

##### Type.

***Holotype***: New Zealand, Nelson Province, growing on bark of *Nothofagusmenziesii* in beech forest along highway between Reefton and Spring Junction, leg. *L. Visch 618*, 14 Jan. 1974 (DUKE156843). ***Isotypes***: (MO2408073!, CHR267040!).

##### Description.

Plants medium-size, yellowish to yellow-green, forming fairly dense mats; stems 2.0–4.0 cm long, in cross-section rounded, the central strand well developed, epidermal cells thick-walled, the parenchyma thin-walled; leaves asymmetrical, lanceolate, plicate and undulate, i.e., transversely folded, concave, long-acuminate; leaves from middle of stem 1.6–2.2 μm long and width measured at widest point 0.6–0.8 μm; apex not denticulate; costae two, weak and thin, not exceeding more than ⅓ of the leaf length; laminal cells asymmetrical, length and width variable but dependent on location: 100–150 × 6–7 μm at midleaf, cell areolation narrow; decurrency of 2–3 rows of rectangular cells forming triangular or wedge-shaped auricles, 150–200 μm long; sporophytes orange, seta reddish below, 2 cm long; capsules cylindrical and inclined; sexual condition unknown (Fig. 6).

*Plagiotheciumfunale* types were recorded from New Zealand, Nelson Province, along highway between Reefton and Spring Junction (MO2408073!, CHR267040!), on bark of *Nothofagusmenziesii* in beech forest (MO2408073!, CHR267040!).

#### ﻿ Plagiothecium cordatum

Taxon classificationPlantaeHypnalesPlagiotheciaceae

G.J.Wolski sp. nov.

C55B20C4-E249-5202-9E90-16F4EEEC0A3A

##### Type.

***Holotype***: New Zealand, Boundary Creek, McKerrow Range, ca 4000 alt., leg. *Colin D. Meurk*, 17 Jan. 1974 (CHR538916!).

##### Description.

Plants small, ascending and julaceous, yellow to yellow-green, with metallic luster, forming dense mats; stems 1.0–2.0 cm long, in cross-section rounded, with a diameter of 220–240 μm, the central strand well-developed, epidermal cells thick-walled, the parenchyma thin-walled; leaves symmetrical, lanceolate, concave, longitudinally folded, imbricate, closely arranged on the stem, those leaves from the middle of stem 1.7–2.0 mm long and the width measured at the widest point 0.7–0.9 mm; the apex acuminate, entire, not denticulate; leaf base cordate-rounded; costae two, weak and thin, extending usually to ½ of leaf length; laminal cells asymmetrical, the length and width variable but dependent on location: 140–165 × 5–7 μm at the apex, 135–160 × 5–7.5 μm at midleaf, 65–100 × 10 μm toward insertion; due to cell width, cell areolation very narrow; decurrency of 3–4 rows of rectangular cells, forming narrow, wedge-shaped auricles, 300 μm long (Fig. 7); sporophytes so far unknown; sexual condition unknown.

*Plagiotheciumcordatum* so far has been recorded from New Zealand, McKerrow Range, Boundary Creek (CHR538916), South Island, Fiordland National Park, Corland Burn, South Branch, 2 km north of Mount Burns (AK352034) and from Macquarie Island, Sawyer Creek (HO610227) (Fig. 7). This species was noted on the south side of southernmost waterfall, on undercut bank at edge of creek (HO610227), on *Nothofagusmenziesii* forest epiphytic on trunk of silver beech (AK352034).

##### Etymology.

The name of this taxon – *Plagiotheciumcordatum* refers to the heart-shaped (Latin: *cor* – heart) base of leaves of this species.

#### ﻿ Plagiothecium semimortuum var. semimortuum

Taxon classificationPlantaeHypnalesPlagiotheciaceae

G.J.Wolski sp. nov.

616F5EAD-81B2-54C0-BED8-6EF731739BE2

##### Type.

***Holotype***: Australia, Victoria, Mt. Stirling at the head of the Delatite River, along steep eastern face, 37°07'S, 146°28'E, alt. 5400 ft., growing on granite rock ledges and crevices along steep eastern face, growing together with *Andreaeaaustralis*, leg. *J. H. Williams 229W*, 8 Mar. 1953 (MEL1016042!). ***Isotype***: (WELT-M28128!).

##### Description.

Plants medium size, ascending and julaceous, yellow-green to dark-green, with metallic luster, forming dense mats; stems 1.0–1.5 cm long, in cross-section rounded, with a diameter of 220–250 μm, the central strand well-developed, epidermal cells thick-walled, 10–15 × 10–12.5 μm, the parenchyma thin-walled, 9.0–14 × 8.0–13 μm; leaves symmetrical, ovate, folded, imbricate, closely arranged on the stem, concave, therefore leaves splitting when flattened, leaves from 1/3 up to 2/3 without protoplasts, those leaves from the middle of the stem 1.6–2.0 mm long and the width measured at the widest point 0.9–1.2 mm; the apex acute, not denticulate; costae two, rather thick and strong, extending usually to ½ of the leaf length, 250–300 μm; laminal cells more or less symmetrical, the length and width variable but dependent on location: 65.0–85 × 10–12.5 μm at apex, 60–90 × 10–12 μm at midleaf, and 65–100 × 15–17.5 μm toward insertion, due to the wide cells, cell areolation loose; decurrency of 4–5 rows of rounded and inflated cells, forming distinct auricles, 250–300 μm long (Fig. 8); sporophytes (immature) with setae reddish at base and yellowish-orange above, 1.5–1.8 cm long; the capsules inclined, 2.0 mm long, operculum long, conical and mammillate; sexual condition unknown.

Plagiotheciumsemimortuumvar.semimortuum so far has been recorded from Australia, near Melbourne (MEL1031370, MEL1016042, CBG50739), Tasmania (HO302794, HO556631, HO133456) and from New Zealand (CHR651872, CHR532442, CHR464681, CHR104940). Specimens of P.semimortuumvar.semimortuum were noted on the ground between plants (CHR651872); on humus between boulders (CHR532442); on shaded rock in exposed southerly sub-alpine herbfields with small scattered low shrubs (CBG50739); within rainforest gully (HO133456); on granite rock ledges and crevices along steep eastern face (MEL1016042, MEL1031370); on alpine heathland (HO556631); crevices in boulder fields (CHR104940); on mat of senescent tussock on vertical side of small valley, *Chionochloapallens*-*Chionochloaaustralis* tussockland with scattered shrubs (CHR 464681). All specimens of P.semimortuumvar.semimortuum have been collected in mountainous areas of Australasia (820–1769 m alt).

##### Etymology.

The name of this species – *Plagiotheciumsemimortuum* (Latin: *semi* – half; *mortum* – dead) refers to the leaves without protoplasts; they are dead even up to half the leaf.

#### ﻿ Plagiothecium semimortuum var. macquariense

Taxon classificationPlantaeHypnalesPlagiotheciaceae

G.J.Wolski var. nov.

CA35B9D8-1D63-5579-80F8-55D62B556F8F

##### Type.

***Holotype***: Australia, Tasmania, Macquarie Island, NW slope of Mt. Haswell, Caroline Cove, 54°44'S, 158°51'E, in *Poafoliosa* dominated vegetation on northwest slopes of Mt. Haswell, 120 m alt., leg. *R. D. Seppelt 15316*, 30 Jan. 1985 (HO610220!).

##### Description.

Plants small, ascending and julaceous, yellow-green, with metallic luster, forming dense mats; stems 0.5–1.0 cm long, in cross-section rounded, with a diameter of 250–280 μm, the central strand well-developed, epidermal cells thick-walled, 7–13 × 6–11 μm, the parenchyma thin-walled, 9–11 × 8–10 μm; leaves symmetrical, narrowly ovate, folded, imbricate, closely arranged on the stem, concave, therefore leaves splitting when flattened, leaves from 1/3 up to 2/3 without protoplasts, those leaves from the middle of stem 1.9–2.2 mm long and the width measured at the widest point 0.9–1.1 mm; the apex acute and denticulate; costae two, rather thick and strong, extending usually to ⅓ of leaf length; laminal cells more or less symmetrical, length and width variable but dependent on location: 112.5–140 × 7.5–10 μm at the apex, 112.5–125 × 7.5–10 μm at midleaf, 88–112 × 15 μm toward insertion; due to the width of the cells, cell areolation tight; decurrency of 4–5 rows of rounded and inflated cells, forming distinct auricles, 200 μm long; sporophytes so far unknown; sexual condition unknown (Figs 9, 10).

Plagiotheciumsemimortuumvar.macquariense so far has been recorded from Australia – Macquarie Island (HO610219, HO610227, HO610220) and mainland Tasmania (HO71698) (Fig. 11). Specimens were noted in *Poafoliosa* (Hook.f.) Hook.f. dominated vegetation on northwest slopes of Mt. Haswell (HO610220); in *Pleurophyllum* Hook.f. dominated plateau herbfield (HO610219); on undercut bank at edge of creek (HO610227). Each specimen of P.semimortuumvar.macquariense was collected in lowland areas (70 to 200 m alt).

**Figure 12.**
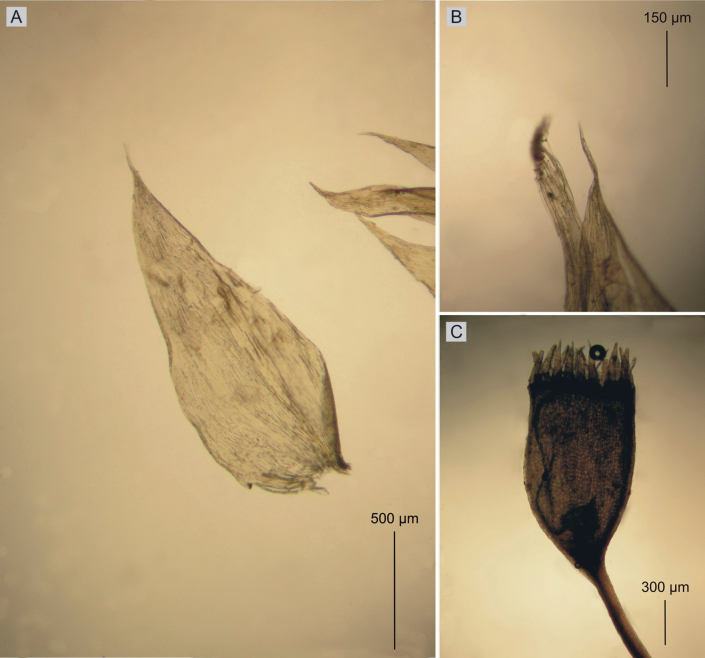
Selected taxonomic features of *Plagiotheciumlucidum***A** shape and dimensions of leaf **B** leaf apex **C** shape and arrangement of capsule (from the type of *P.lucidum* PC0132689!, PC0132690!, photo. G. J. Wolski, November 18, 2021).

##### Etymology.

The name of this variety — Plagiotheciumsemimortuumvar.macquariense — refers to Macquarie Island (Australia, Tasmania), from which the plant was first recorded, and where the holotype (HO610220) was collected.

### ﻿Key to the species of *Plagiothecium* known from Australasia

**Table:** 

1	Decurrency composed of rectangular, non-inflated cells, forming wedge-shaped groups (Fig. 10F, G)	**2**
–	Decurrency composed of spherical, inflated cells, forming distinct auricles (Fig. 10A–E)	**4**
2	Leaves with long-acuminate apex (Fig. 12)	***P* . *lucidum***
–	Leaves with acute to short-acuminate apex	**3**
3	Leaves asymmetric, lanceolate, transversely folded (Fig. 10F)	***P* . *funale***
–	Leaves symmetric, julaceous on the stem and imbricate (Fig. 10G), longitudinally folded	***P* . *cordatum***
4	Leaves up to 2/3 devoid of protoplasts	**5**
–	Leaves without protoplast-free areas	**6**
5	Cells in middle part of leaf short and broad (60–90 × 10–12 µm) making the cellular areolation loose, specimens growing on mountains	***P* . *semimortuum*var.semimortuum**
–	Cells from middle part of leaf long and narrow (112.5–125 × 7.5–10 µm) which makes the cell areolation tight, specimens recorded in lowlands	***P* . *semimortuum*var.macquariense**
6	Plants complanate-foliate; leaves asymmetrical (Fig. 10B)	**7**
–	Plants julaceous; leaves symmetrical (Fig. 10A); apex not serrate; cell areolation narrow (100–140 × 7.5–10 µm)	***P* . *novae-seelandiae*var.brotheri**
7	Leaves quite short and wide (1.7–2.2 × 1.0–1.5 mm), clearly asymmetrical (Fig. 10B); apex serrate	***P* . *novae-seelandiae*var.novae-seelandiae**
–	Leaves concave, long and wide (2.5–2.6 × 1.0–1.2 mm), asymmetric or slightly asymmetrical (Fig. 10C); apex not serrate	***P* . *lamprostachys***

## Supplementary Material

XML Treatment for Plagiothecium lamprostachys

XML Treatment for Plagiothecium novae-seelandiae var. novae-seelandiae

XML Treatment for Plagiothecium novae-seelandiae var. brotheri

XML Treatment for Plagiothecium funale

XML Treatment for Plagiothecium cordatum

XML Treatment for Plagiothecium semimortuum var. semimortuum

XML Treatment for Plagiothecium semimortuum var. macquariense

## ﻿Additional information

### Author ORCIDs

Grzegorz J. Wolski https://orcid.org/0000-0003-1480-8003

Mikołaj Latoszewski https://orcid.org/0009-0003-5228-210X

D. Christine Cargill https://orcid.org/0000-0001-8390-3245

### Data availability

All of the data that support the findings of this study are available in the main text.
